# Comparison of the efficacy of tenofovir and adefovir in the treatment of chronic hepatitis B: A Systematic Review

**DOI:** 10.1186/1743-422X-8-111

**Published:** 2011-03-09

**Authors:** Shu-Shan Zhao, Lan-Hua Tang, Xia-Hong Dai, Wei Wang, Rong-Rong Zhou, Li-Zhang Chen, Xue-Gong Fan

**Affiliations:** 1 Department of Infectious Diseases, Xiangya Hospital, Central South University, Changsha, Hunan, PR China; 2 Eight-Year Program, Xiangya School of Medicine, Central South University, Changsha, Hunan, PR China; 3 Department of Spine Surgery, Xiangya Hospital, Central South University, Changsha, Hunan, PR China; 4 Department of Neurology, Xiangya Hospital, Central South University, Changsha, Hunan, PR China; 5 Department of Gastroenterology, Third Xiangya Hospital, Central South University, Changsha, Hunan, PR China; 6 School of Public Health, Central South University, Changsha, Hunan, PR China

## Abstract

Chronic viral hepatitis B remains a global public health concern. Currently, several drugs, such as tenofovir and adefovir, are recommended for treatment of patients with chronic hepatitis B. tenofovir is a nucleoside analog with selective activity against hepatitis b virus and has been shown to be more potent in vitro than adefovir. But the results of trials comparing tenofovir and adefovir in the treatment of chronic hepatitis B were inconsistent. However, there was no systematic review on the comparison of the efficacy of tenofovir and adefovir in the treatment of chronic hepatitis B. To evaluate the comparison of the efficacy of tenofovir and adefovir in the treatment of chronic hepatitis B we conducted a systematic review and meta-analysis of clinical trials. We searched PUBMED, Web of Science, EMBASE, CNKI, VIP database, WANFANG database, the Cochrane Central Register of Controlled Trials and the Cochrane Database of Systematic Review. Finally six studies were left for analysis which involved 910 patients in total, of whom 576 were included in tenofovir groups and 334 were included in adefovir groups. At the end of 48-week treatment, tenofovir was superior to adefovir at the HBV-DNA suppression in patients[RR = 2.59; 95%CI(1.01-6.67), P = 0.05]. While there was no significant difference in the ALT normalization[RR = 1.15; 95%CI(0.96-1.37), P = 0.14], HBeAg seroconversion[RR = 1.32; 95%CI(1.00-1.75), P = 0.05] and HBsAg loss rate[RR = 1.19; 95%CI(0.74-1.91), P = 0.48]. More high-quality, well-designed, randomized controlled, multi-center trails are clearly needed to guide evolving standards of care for chronic hepatitis B.

## 1. Introduction

Chronic hepatitis B virus (HBV) infection is a serious global public health problem associated with cirrhosis, liver failure and hepatocellular carcinoma (HCC) [1]. Of the two billion people who have been infected, more than 350 million have chronic hepatitis [2]. Several major advances in the treatment of chronic hepatitis B(CHB) have been made over the last several years. Currently, several drugs are recommended for treatment of patients with CHB. These drugs can be divided into two main groups based on their mechanism of action, namely immunomodulatory drugs like alpha interferons and antiviral drugs including lamivudine(LAM), telbivudine(LdT), entecavir(ETV), adefovir(ADV), and tenofovir(TDF)[3].

Adefovir dipivoxil, an acylic phosphonate, is a nucleotide analog licensed for the treatment of HBV, which is active against both negative and positive HBeAg [4,5]. Furthermore, it has been shown that ADV has an excellent activity against wild-type as well as LAM-resistant HBV strains [6,7]. However, ADV has low HBeAg seroconversion rate which was only 12%, less potency toward HBV DNA suppression and a few drawbacks including nephrotocicity for those who are at risk for renal dysfunction [5]. Meanwhile at the licensed dose of 10 mg/day ADV is not a highly potent drug [8]. But the advantage of ADV was the low rates of resistance. In contrast to LAM, after 2 and 4 years of ADV treatment only 3%, 18% of patients exhibit resistance respectively [9]. In addition, both the ADV mutations, N236T and A181V, are susceptible to LAM. So ADV is a safe and effective treatment option for HBV infection.

Tenofovir disoproxil fumarate (TDF), a new nucleotide analogue licensed in 2008 for the treatment of HBV infections in Europe and the United States [10], also exhibits an efficacious activity against wild type and LAM-resistant HBV, both *in vitro*[11] and *in vivo*[12,13]. Randomized clinical trials have shown that TDF is more potent than ADV in inhibiting HBV replication in patients with chronic hepatitis [14]. The same study showed the efficacy of TDF in patients with a LAM-resistant virus and the absence of resistant mutations after 48weeks of therapy. Besides, it has shown antiviral efficacy against a broad spectrum of viral infections, including human immunodeficiency virus (HIV-1)[15].

Treatment of CHB with TDF versus ADV have been reported in six independent studies [14,16-19], two of them [16,18] on HIV-HBV coinfected patients and another two of them on patients with LAM-resistant [17,19]. These studies suggest a more promising result using TDF than ADV. However, there are important differences among these trials in study design, patient populations and patient status. The aims of this review are to summarize their findings and assess the impact on our management of the disease.

## 2. Methods

### 2.1. Search strategy

We searched the following databases until August 2010: PUBMED (from 1990 to August 2010), Web of Science (from 1990 to August 2010), EMBASE (from 1990 to August 2010), CNKI (National Knowledge Infrastructure) (from 1990 to August 2010), VIP database (from 1990 to August 2010), WANFANG database (from 1990 to August 2010), the Cochrane Central Register of Controlled Trials and the Cochrane Database of Systematic Review. Of these databases, CNKI, WANFANG and VIP databases provide literatures in Chinese. The search process was designed to find initially all trials involving terms: "Hepatitis B", "tenofovir", "adefovir"(and multiple synonyms for each term). Reference lists from retrieved documents were also searched. Computer searches were supplemented with a manual search. Search results were downloaded to a reference database and further screened. Two authors (Shushan Zhao and Lanhua Tang) independently screened all citations and abstracts identified by the search strategy to identify potentially eligible studies.

### 2.2. Types of studies

All relevant clinical trials will be included, irrespective of language, or blinding. Observational studies will be excluded except for their report on harms.

### 2.3. Types of participants

Male or female patients, of any age or ethnic origin, who have CHB, defined as CHB virus infection with evidence of hepatitis (alanine aminotransferase (ALT) elevation of at least one and a half times the upper limit of normal range) and of viral replication (detectable hepatitis B virus DNA by DNA hybridisation method or polymerase chain reaction (PCR)), will be included. Patients with decompensated liver disease, hepatocellular carcinoma, prior liver transplantation and concomitant renal failure were excluded.

### 2.4. Types of interventions

The comparisons will include TDF versus ADV.

### 2.5. Types of outcome measures

Proportion of patients with HBV-DNA level under 1000 copies/ml, ALT normalization rate, HBeAg seroconversion rate, HBsAg loss rate.

### 2.6. Data extraction

Data was extracted independently by both authors (Shushan Zhao and Lanhua Tang) using a pre-designed data extraction form and the information subsequently was entered into Review Manager (RevMan 5.0). Information was extracted on data source; eligibility; methods; participants (age range, exclusion criteria, sample size, gender); interventions; and results. We resolved any discrepancies between the extracted data by discussion, and, if required, referral to the third author (Rongrong Zhou). Where data were not clear or not presented by the author in the publication, we attempted to contact the trial author for further details. Data was extracted on the methodological domains relevant to minimising bias and random error in the analysis of trials by using the Cochrane methods for assessing risk of bias and GRADE methods [20]. Specifically, we assessed study limitations by evaluating the method of randomisation, allocation concealment, blinding, analysis by intention to screen, free of selective reporting and so on. As perGRADE [20], we further assessed the quality of evidence with regard to limitations in designs, inconsistency, indirectness, imprecision, and other potential sources of bias, such as publication and reporting bias. GRADE criteria were then applied to downgrade the quality of evidence when indicated on an outcome specific basis. The quality of evidence for an individual outcome was ultimately rated as high, moderate, low, or very low.

### 2.7. Quality assessment

Quality of the trials was assessed using the QUOROM guidelines as well as using the Jadad scale. These prompted downgrading of the overall quality of evidence for all individual outcomes.

### 2.8. Data analysis

Data analysis was carried out with the use of Review Manager Software 5.0(Cochrane Collaboration, Oxford, United Kingdom). For each eligible study, dichotomous data were presented as relative risk (RR), which is the probability that a member of an exposed group will develop a disease relative to the probability that a member of an unexposed group will develop that same disease, and continuous outcomes were presented as weighted mean difference (WMD), which is calculated as the difference between the mean value in the treatment and control groups, both with 95% confidence intervals (CI). Meta-analysis was performed using fixed-effect or random-effect methods, depending on the absence or presence of significant heterogeneity. Statistical heterogeneity between trials was evaluated by the chi-square and I-square (I^2^) tests, with significance set at P < 0.10. In the absence of statistically significant heterogeneity, the fixed-effect method was used to combine the results. When heterogeneity was confirmed (P < 0.10), the random-effect method was used. Additionally, sensitivity analysis should be carried out if low quality trials were included. The overall effect was tested using z scores calculated by Fisher's z' transformation, with significance set at P < 0.05.

## 3. Results

**Figure 1:**
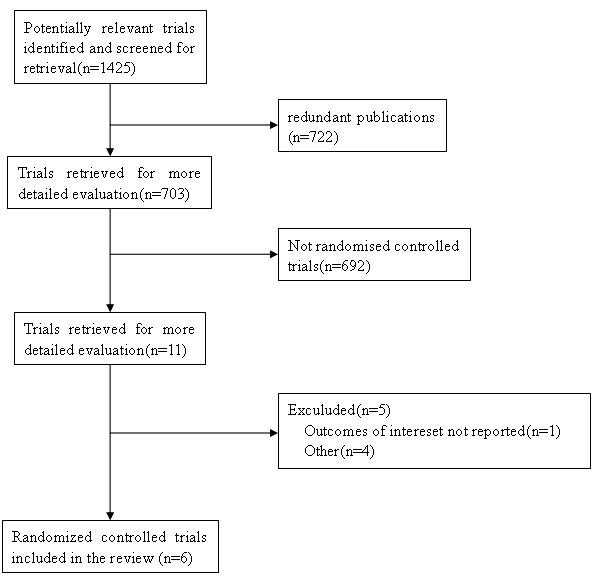
**Flow diagram of the studies identified**.

We searched relevant literatures, and finally a total of 1425 studies identified by the searches(PUBMED:161; Web of Science:404; EMBASE:844; CNKI:3; VIP database:1; WANFANG database:1; the Cochrane Central Register of Controlled Trials and the Cochrane Database of Systematic Review:11). By scanning titles and abstracts, 722 redundant publications, review, and meta-analysis were excluded. After referring to full texts, 697 studies that did not satisfy the inclusion criteria were removed from consideration. Six studies [14,16-19] were left for analysis which involved 910 patients in total, of whom 576 were included in TDF groups and 334 were included in ADV groups (Figure 1). According to treatment period, we divided the studies into two subgroups: LAM-resistance group and HBV-HIV coinfected group. In addition, all studied populations with comparable baseline characteristics between TDF groups and ADV groups. Of the six trials, all were published in English. The detailed information of included trials was summarized in Table 1 and Table 2. With the exception of the study by van Bommel *et al *[19]., which was not referred, all were multicentred. However only four studies [14,17,18] were multicentred involving sites in different countries. The lastest one [14] was a phase □ trial comparing TDF with ADV. Of all trials, three were random double-blind trials. The treatment duration was more than 48 weeks and the end points were assessed at week 48. All six trials contained patients of similar demographics in terms of age and sex distribution(Table 1). The majority of patients were in their third or fourth decade of age and there was a male preponderance in all studies. Two studies [17,19] included LAM-resistance patients and two studies [16,18] included HIV-HBV coinfetion patients. Different HBV-DNA assays were used in these studies and thus the HBV-DNA levels may not be directly comparable between studies.

**Table 1:** Summary of study design of the six trials

Study	Marcellin Study 102 [14]	Marcellin Study 103 [14]	Lacombe [16]	Hann [17]	Peters [18]	van Bommel [19]
Study location	Worldwide	Worldwide	French multi-centre	Asian-Americans	Worldwide	N/A
Study design	randomized,double-blind	randomized,double-blind	non-randomized, open label	quasi-randomized, open-label	prospective randomized, double-blind	Nonrandmoized,open-label
Grade	5	5	2	3	5	2
Treatment options	Tenofovir 300 mg/d vs Adefovir 10 mg/d	Tenofovir 300 mg/d vs Adefovir 10 mg/d	Tenofovir 300 mg/d vs Adefovir 10 mg/d	Tenofovir 300 mg/d vs Adefovir 10 mg/d	Tenofovir 300 mg/d vs Adefovir 10 mg/d	Tenofovir 300 mg/d vs Adefovir 10 mg/d
Male	193(77%) vs. 97(78%)	119(68%) vs. 64(71%)	54(96.4%) vs. 28(26.6%)	37(85%) vs. 49(76%)	24 (89%) vs. 24 (96%)	32(91.4%) vs. 14(77.8%)
Mean age, years	44 ± 10.6 vs. 43 ± 10	34 ± 11 vs. 34 ± 12	42.3 vs. 41.5	49 vs. 45	40 vs. 47	47 ± 2 vs. 45 ± 3.7
HBeAg-positive (%)	0:0	1:1	50(89.3%) vs. 25(86.2%)	33(75%) vs. 55(84%)	23 (85%) vs. 20 (80%)	31(89%) vs16( 89%)
Treatment (weeks)	48 weeks	48 weeks	48 weeks	48 weeks	48 weeks	72 to 130 weeks vs. 60 to 80 weeks
Follow-up (weeks)	N/A	N/A	N/A	48 weeks	91 weeks vs. 81 weeks	N/A
Status	N/A	N/A	Coinfect HIV	lamivudine-resistant	Coinfect HIV	lamivudine-resistant

**Table 2:** Methodological quality of randomised controlled trials

Study	Adequate sequence generation	Allocation concealment	Blinding	Incomplete outcome data addressed	Free of selective reporting	Free of other bias
Marcellin Study 102 [14]	yes	yes	yes	yes	yes	yes
Marcellin Study 103 [14]	yes	yes	yes	yes	yes	yes
Lacombe [16]	no	no	no	yes	yes	unclear
Hann [17]	unclear	unclear	unclear	yes	unclear	unclear
Peters [18]	yes	yes	yes	yes	yes	yes
van Bommel [19]	no	no	no	yes	yes	unclear

In the trial by van Bommel *et al *[19], results showed that TDF coped more effectively with the LAM-resistant virus population than ADV at week 48 which were in accordance with results from previous studies demonstrating a HBV DNA decline at week 24. In this trial, TDF is superior to ADV considering the fact that in all TDF-treated patients HBV DNA became negative compared with only approximately 44% of the ADV group. And ALT levels normalized more rapidly in the TDF group than in the ADV group. Interestingly, 5 of the TDF-treated patients lost HBsAg. No major clinical side effects were reported during treatment with either TDF or ADV. In the trial by Hann *et a*[17]*l*, results showed that after 6 months treatment, HBV DNA levels were significantly reduced for the TDF group than for the ADV group. After 12 months on therapy, mean HBV DNA levels were reduced to 1.5 ± 1.0 log copies/ml for the TDF group and 4.3 ± 2.2 log copies/ml for the ADV group and the difference was significant which accorded with the result of Bommel's. However ALT normalization and HBeAg loss at 12 months showed no significant difference between two group.

In the study by Peters *et al *[18], either ADV or TDF treatment resulted in clinically significant reductions in serum HBV DNA levels in coinfected patients. TDF was not inferior to ADV according to the HBV DNA suppression. At week 48, 25% of subjects on ADV and 36% of subjects on TDF normalized serum ALT, but these numbers were not statistically significant either.

But in the study by Lacombe *et al *[16], the HBV-DNA decay was more significant in patients treated with TDF than with ADV, even after adjustment for baseline HBV viral load. And undetectability (HBV-DNA < 200 copies/mL) was reached by 66% and 28% patients treated with TDF and ADV, respectively (p = 0.04). However similar rates of HBeAg seroconversion and HBsAg loss were found between two groups.

The study by Marcellin *et al *[14] include two trials: study 102 (HBeAg-negative patients) and study 103 (HBeAg-positive patients). In both studies, a significantly greater proportion of patients who received TDF than patients who received ADV reached the HBV DNA level of less than 400 copies/ml. Among the HBeAg-negative patients, 93% of all the patients who received TDF had a plasma HBV DNA level of less than 400 copies/ml by week 48 while 63% of all the patients who received ADV had(p < 0.01). Among HBeAg-positive patients, 76% of patients who received TDF had an HBV DNA level of less than 400 copies/ml at week 48 while 13 of patients who received ADV had(p < 0.01). In Study 102, similar proportions of patients in the two treatment groups had normalized alanine aminotransferase levels at week 48, whereas in Study 103, a significantly greater proportion of patients in the TDF group had normalized alanine aminotransferase levels.

Despite the differences in types of patient population, study design and patients' status, the six trials appear to demonstrate that TDF is superior to ADV in HBV-DNA decay. However there are different results on ALT normalization. So a meta-analysis is necessary to combine the results of those six studies.

**Figure 2:**
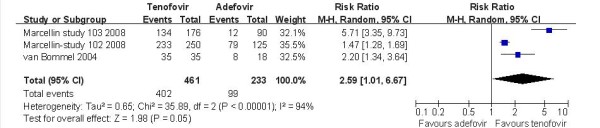
**Effect of TDF vs. ADV on HBV-DNA suppression at week 48**.

**Figure 3:**
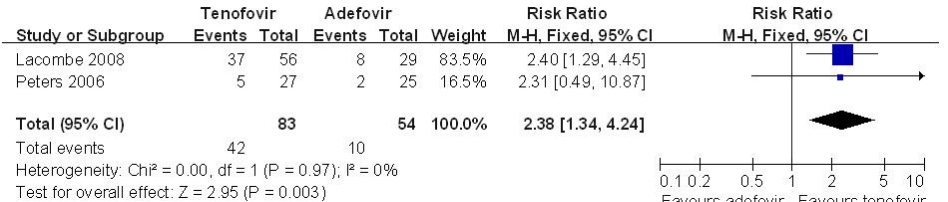
**Effect of TDF vs. ADV on HBV-DNA suppression in HBV-HIV subgroup at week 48**.

Only three trials [14,19] demonstrated the HBV-DNA level decreases below 400 copies/ml. According to chi-squared statistic and I square(I^2^), heterogeneity was assessed and had significant differences[Tau^2 ^= 0.65; Chi^2 ^= 35.89, df = 2 (P < 0.00001); I^2 ^= 94%]. A summary estimate of the relative risk of TDF versus ADV by use of a random-effects approach. The results of the three trials showed HBV-DNA level below 400 copies/ml in the TDF group was 87.2%, compared to 42.5% in the ADV group at week 48. And the efficacy of HBV-DNA suppression rates in TDF group was higher than ADV group[RR = 2.59, 95%CI(1.01-6.67), P = 0.05](Figure 2). However, the conclusion was changed when the low quality trial [19] was removed[RR = 2.58, 95% CI(0.56-14.56), P = 0.21]. The quality of evidence for this effect was judged low (Table 3). Just one study [19] demonstrated the HBV-DNA level decreases below 400 copies/ml in LAM-resistance subgroup. The result showed the HBV-DNA suppression rates in TDF group was higher than ADV group[RR = 2.20, 95%CI(1.34-3.64), P = 0.002]. Two studies [16,18] demonstrated the HBV-DNA level decreased below 200 copies/ml in HBV-HIV coinfected subgroup. The results showed HBV-DNA level below 200 copies/ml for in the TDF group was 50.6%, compared to 18.5% in the ADV group at week 48. And the HBV-DNA suppression rates in TDF group was higher than ADV group[RR = 2.38, 95%CI(1.34-4.24), P = 0.003](Figure 3). The quality of evidence for this effect was judged moderate.

**Table 3:** Methodological quality of randomised controlled trials

Outcomes	Illustrative comparative risks* (95% CI)		Relative effect (95% CI)	No of Participants (studies)	Quality of the evidence (GRADE)†
	Assumed risk (per 1000)	Corresponding risk (per 1000)			
	Adefovir	Tenofovir			
HBV-DNA suppression	425	1000(429 to 1000)	RR 2.59 (1.01 to 6.67)	694(3 studies)	**low⊕⊕▯▯**
HBV-HIV coinfected subgroup	185	440(248 to 784)	RR 2.38 (1.34 to 4.24)	137(2 studies)	**moderate⊕⊕⊕▯**
ALT normalization	634	729(609 to 869)	RR 1.15 (0.96 to 1.37)	768(5 studies)	**low⊕⊕▯▯**
lamivudine-resistance subgroup	554	731(554 to 964)	RR 1.32 (1.00 to 1.74)	132(2 studies)	**low⊕⊕▯▯**
HBeAg seroconversion	140	167(104 to 267)	RR 1.19 (0.74 to 1.91)	387(3 studies)	**low⊕⊕▯▯**
HBsAg loss	0	0	RR 5.74 (0.32 to 102.59)	615(2 studies)	**high⊕⊕⊕⊕**

*The corresponding risk (and its 95% CI) is based on the assumed risk in the entecavir group and the relative effect of the lamivudine (and its 95% CI).

**†**High quality: Further research is very unlikely to change our confidence in the estimate of effect.

Moderate quality: Further research is likely to have an important impact on our confidence in the estimate of effect and may change the estimate. Low quality: Further research is very likely to have an important impact on our confidence in the estimate of effect and is likely to change the estimate. Very low quality: We are very uncertain about the estimate.

**Figure 4:**
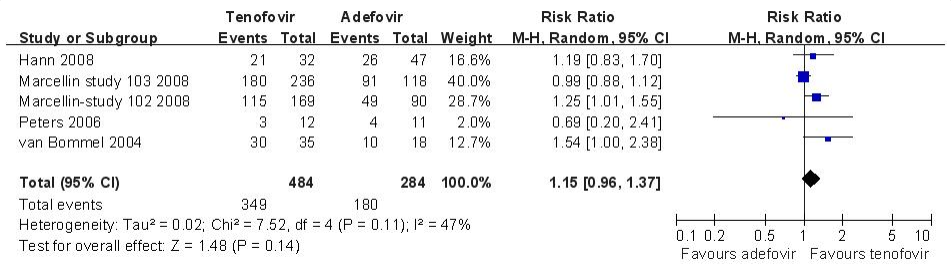
**Effect of TDF vs. ADV on ALT normalization at week 48**.

**Figure 5:**
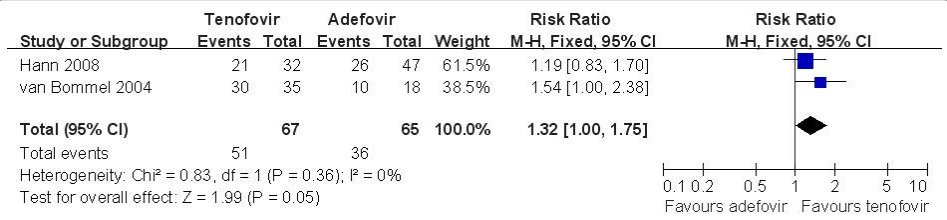
**Effect of TDF vs. ADV on ALT normalization in lamivudine-resistance subgroup at week 48**.

Five studies [14,17-19] demonstrated the ALT normalization rate in this subgroup. According to chi-squared statistic and I square (I^2^), a random-effects approach was used to summary estimate the relative risk of TDF versus ADV. The results of the five trials showed normalization rates for ALT in the TDF group as 72.1%, compared to 63.3% in the ADV group at week 48. And the biochemical response rates in TDF group was similar with the ADV group [RR = 1.15, 95%CI(0.96-1.37), P = 0.14](Figure 4). The quality of evidence for this effect was judged low. The result was unchanged when low quality trials [17,19] were removed[RR = 1.08, 95%CI(0.87-1.33), P = 0.49]. The quality of evidence for this effect was judged moderate. In the LAM-resistance subgroup, a fix-effects approach was used to summary estimate the relative risk of TDF versus ADV and the efficacy of TDF versus ADV on ALT normalization rates is also similar[RR = 1.32, 95%CI(1.00-1.74), P = 0.05] (Figure 5). The quality of evidence for this effect was judged low.

**Figure 6:**
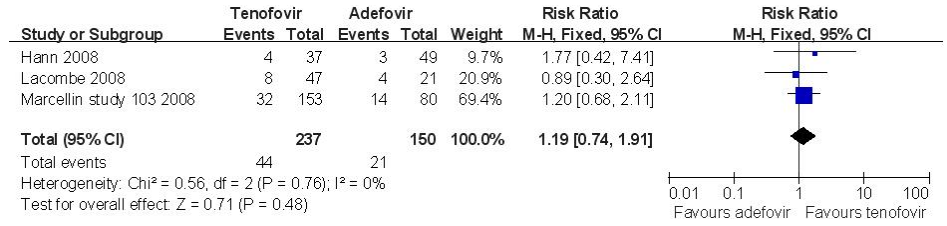
**Effect of TDF vs. ADV on HBeAg seroconversion rate at week 48**.

Three trials [14,16,17] demonstrated the HBeAg seroconversion rate in this subgroup. According to chi-squared statistic and I square(I^2^), heterogeneity was assessed and not found to be a concern[Chi^2 ^= 0.00, df = 1 (P = 0.97); I^2 ^= 0%]. The results of the three studies showed the HBeAg seroconversion rate for the TDF group was 18.6% while the ADV group response rate was 14.0%. The difference of HBeAg seroconversion rates at week 48 between the two group was similar[RR = 1.19, 95%CI (0.74-1.91), P = 0.48] (Figure 6). The quality of evidence for this effect was judged low. Two trials demonstrated the HBsAg loss rate in this subgroup and the difference of HBsAg loss rate between the two group was similar[RR = 5.74, 95%CI (0.32-102.59), P = 0.23]. The quality of evidence for this effect was judged high (Table 3).

### Toleraility and safety of tenofovir and adefovir

The most frequently reported adverse events in TDF-treatmented patients, which included headache, nasopharyngitis, fatigue, diarrhea, upper abdominal pain, and so on, were those normally associated with ADV except nausea. In Marcellin's trial, it's report that nausea was the only adverse event that consistently occurred more frequently in the group of patients who received TDF than in the group of patients who received ADV. Among the cases of nausea that were considered to be related to TDF, nausea was mild except for one case of grade 2 (moderate) nausea. Despite presence of these adverse effects, rate of withdrawal from both treatments was rare in all studies. In both studies, similar proportions of patients in the two treatment groups had a serious adverse event, and few events were considered to be related to the study drug. The frequency of alanine aminotransferase flares during treatment was also similar in the two groups. These was no death associated with the treatment, or liver decompensation.

## 4. Dicussion

There have been several meta-analysis comparing drugs in the treatment of chronic hepatitis B [21,22], but no comparison of the efficacy of TDF and ADV have been performed. So we conducted this meta-analysis to compare antiviral efficacy of TDF and ADV in the treatment of CHB; despite variations in study design among the six studies, the results were nearly similar, which showed that at the end of 12 months treatment, TDF was better than ADV at the HBV-DNA suppression, while there was no significant difference in the ALT normalization, HBeAg seroconversion and HBsAg loss rate. Meanwhile there are similar results in the ALT normalization in the LAM-resistance and suppression of viral replication in HIV-HBV coinfected subgroup. The inter-heterogeneity was not statistically significant(P > 0.1). There were on changes about effect when a random-effect model was used. Interestingly, the result of HBV-DNA suppression was changed when the low quality [19] trial was removed[RR = 2.58, 95%CI(0.56-14.56) P = 0.21]. But the confidence intervals became wider, so we think that the findings should be reinterpreted when more researches done. Adverse events related to TDF and ADV did occur but none appeared to be lifethreatening. So this meta-analysis showed that TDF was a more potent therapy for the treatment of chronic HBV infection than ADV. However, TDF was just effective in suppressing HBV-DNA levels. Considering its favorable long-term safety record in patients with HIV-1 infection, TDF should be used for the treatment of chronic HBV infection.

While the results of this meta-analysis were promising, there are questions that remain unresolved. Peters and Lacombe's study [16,18] have been the only two trials to compare efficacy of HBV-DNA suppression in HIV coinfected patients, however those two trials have a small sample size and different study location. van Bommel and Hann's trials [17,19] have been the only two studies to compare efficacy of ALT normalization in LAM-resistance patients, however both trials have a low Jadad score and small sample size. Although this meta-analysis showed TDF was superior to ADV on HBV DNA suppression in HIV-coinfected subgroup, these two trials had different Jadad score. And when the low quality [16] trials was removed, the conclusion was changed [RR = 2.31, 95%CI (0.49-10.87), P = 0.29]. So is TDF really more efficacious than ADV on HBV-DNA suppression in the HIV coinfected patients and similar with ADV on ALT normalization in the LAM-resistance subgroup? More solid data is needed in the future. Another unresolved issue is related to the HBeAg seroconversion and HBsAg loss to TDF and ADV therapy in HBV infection. The studies so far have provided no evidence of efficacy in terms of HBeAg seroconversion and HBsAg loss. Meanwhile, follow-up of patients will be needed to detect delayed viral suppression or relapse. More importantly, the clinical outcomes in terms of liver-related complications, mortality and hepatocellular carcinoma should be assessed [23]. Besides there are different outcomes on HBV-DNA level in the six trials and it made difficult to extract data, so it is necessary to make a basic uniform standards for all trails outcome. After all, more high-quality, well-designed, randomized controlled, multi-center trails comparing TDF and ADV in LAM-resistance or HIV coinfected patients would be needed.

There are several methodological limitations in the trials that have been reviewed warrant some discussion. First, only three studies were double-blinded. Although the intervention is easy to blind, there are several trials didn't use. However, it is unlikely that the lack of blinding could affect the outcomes assessed [24]. Second, as LAM-resistance and HIV-HBV coinfected patients were studied in two of the six included trials respectively, it is likely that our meta-analysis included different patient groups. So the multiple subgroup analyses are performed to detect the efficacy of TDF and ADV in the treatment of CHB. Third, three trials didn't describe the method used to generate the allocation sequence. Despite these potential sources of bias, randomization was adequate in the six trials as shown by the baseline equivalency of treatment groups. Finally, the different HBV DNA assays used in the different trials may also have caused additional variability in the sensitivity of HBV DNA detection and thus in the estimate of efficacy.

Another potentially important limitation of meta-analysis is publication bias, the fact that not all research is published. Compared to positive studies, negative studies may be less likely to be published and more likely to take longer to be published, which can affect the validity of meta-analysis [25]. One commonly used method to detect publication bias is the 'funnel plot,' which is a scatter plot that displays the relationship between the weight of the study and the observed effect. In principle, larger studies should display less variability of the treatment effects. Asymmetric appearance, especially due to the absence of smaller negative studies, can suggest unpublished data. However, neither Egger's test nor funnel plots showed evidence for publication bias.

In summary, the results of this meta-analysis indicate that a twelve-month TDF treatment was superior to ADV in inhibiting HBV replication in CHB patients. But there was no significant difference in the ALT normalization, HBeAg seroconversion and HBsAg loss rate. More high-quality, well-designed, randomized controlled, multi-center trails that are adequately powered are clearly needed to guide evolving standards of care for CHB.

## Competing interests

The funding source had no influence on study design, in the collection, analysis, and interpretation of the data, in the writing of the manuscript, or in the decision to submit the manuscript for publication. The contents are solely the responsibility of the authors and do not necessarily represent the views of the funding source.

## Authors' contributions

XGF conceived the study, provided fund supporting and revised the manuscript critically for important intellectual content. SSZ, RRZ and LHT made substantial contributions to its design, acquisition, analysis and interpretation of data. LZC, WW and XHD participated in the design, acquisition, analysis and interpretation of data. All authors contributed equally to this manuscript. All authors read and approved the final manuscript.
